# Complete Genomic Analysis of the Novel Phage BUCT-3589, Infecting Klebsiella pneumoniae

**DOI:** 10.1128/mra.01223-22

**Published:** 2023-03-02

**Authors:** Xiaoxuan Zhang, Yanze Mi, Shan Xu, Lihua Song, Huahao Fan, Mengzhe Li, Yigang Tong

**Affiliations:** a College of Life Science and Technology, Beijing University of Chemical Technology, Beijing, China; Queens College Department of Biology

## Abstract

We report the complete genome sequence of the phage BUCT-3589, infecting multidrug-resistant Klebsiella pneumoniae 3589. It is a new member of the genus *Przondovirus* in the family *Autographiviridae* and possesses a double-stranded DNA (dsDNA) genome of 40,757 bp with 53.13% GC content. The genome sequence will support its use as a therapeutic agent.

## ANNOUNCEMENT

Klebsiella pneumoniae, a Gram-negative pathogen, can cause various infectious diseases, including urinary tract infections, bacteremia, pneumonia, and liver abscesses (1). With the widespread use of antibiotics, multidrug-resistant K. pneumoniae strains have increased annually. Phages have been proposed as a promising treatment for multidrug-resistant bacteria, as they can specifically target and kill the bacteria with few side effects (2).

We used K. pneumoniae 3589, a carbapenem-resistant Klebsiella pneumoniae (CRKP) strain, as the host to isolate phages using the double-layer agar method (3), and suspected plaques were purified at least three times. The morphology of the phages was observed by transmission electron microscopy (TEM). First, the phages were ultracentrifuged and purified by sucrose density gradient centrifugation (4). Then, 30 μL of the purified phage suspension (10^10^ PFU/mL) was dropped onto carbon-coated copper grids for 10 min, which were then negatively stained with 2% (wt/vol) uranylacetate for 90 s, followed by examination using TEM at 100 kV (Hitachi H-7650; Tokyo, Japan) (5).

Genomic DNA of the phage was extracted using the proteinase K-SDS method (6). Whole-genome sequencing was performed using the Illumina NovaSeq 6000 platform (Thermo Fisher Scientific, MA, USA). A sequencing library was constructed using the Illumina paired-end (PE) cluster kit (Illumina, USA). After cluster generation, the DNA library was sequenced on the Illumina platform, and 150-bp paired-end reads were generated. Trimmomatic V0.36 was used to filter the low-quality sequences (7). SPAdes V3.13.0 was employed to assemble the complete genomic sequence (8). Default parameters were used for all software. A total of 4,542,557 reads were obtained by sequencing. After filtering, 4,441,861 reads remained. After assembly, a total of 11,727 contigs (*N*_50_, 26,312 bp) were obtained. The whole-genome coverage of bacteriophage BUCT-3589 was 100%. The Nucleotide Basic Local Alignment Search Tool (BLASTn) was used to compare the nucleotide sequences of different phage genomes. Putative open reading frames (ORFs) were annotated using RAST (https://rast.nmpdr.org/). Protein functions were predicted using NCBI’s Protein Basic Local Alignment Search Tool (BLASTp).

**FIG 1:**
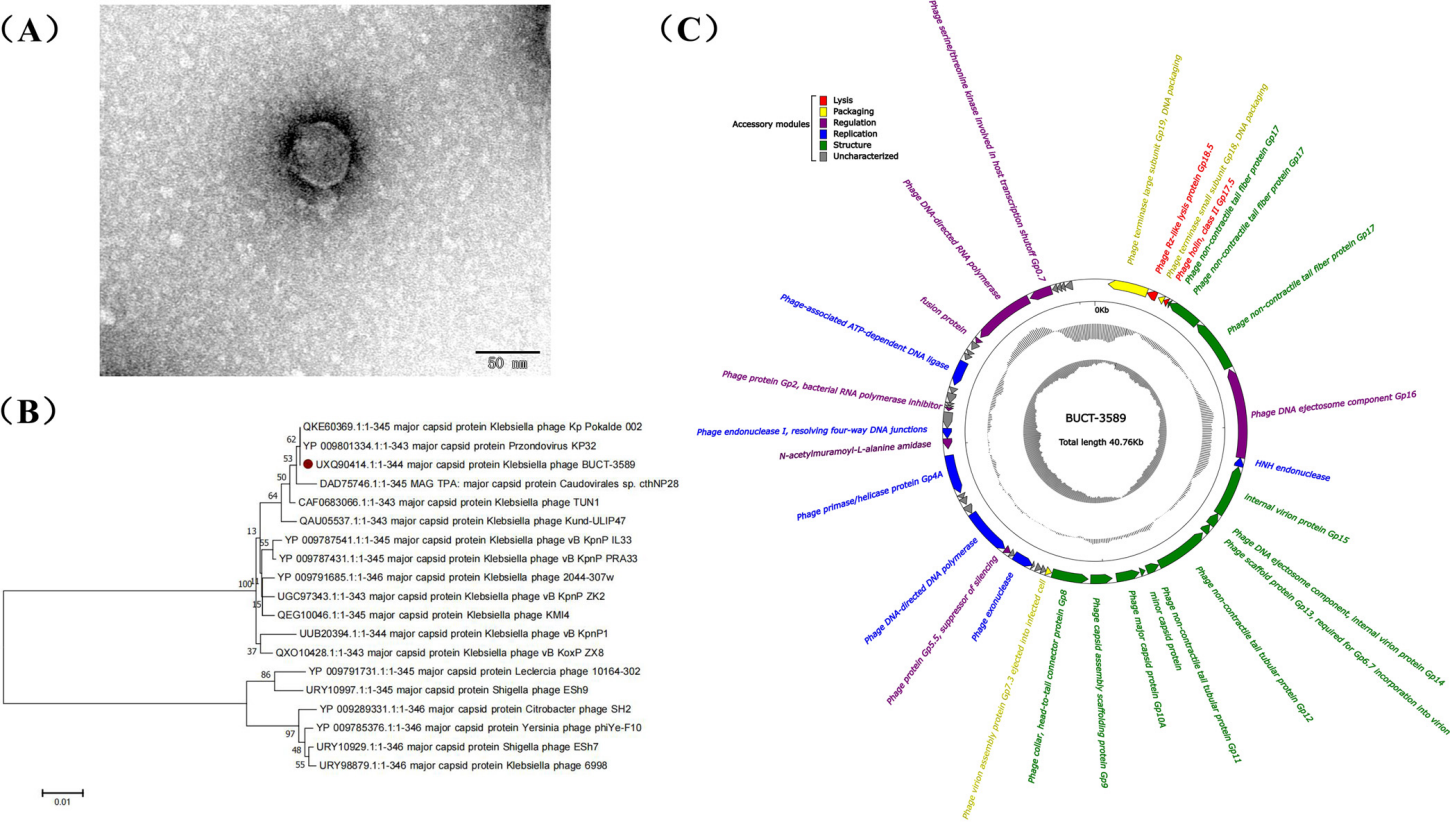
Overview of bacteriophage BUCT-3589. (A) TEM image of phage BUCT-3589. (B) A phylogenetic tree was constructed using the neighbor-joining method with MEGA V7.0 software based on the classic amino acid sequences, major capsid proteins. (C) Genome map of bacteriophage BUCT-3589. The outermost circle represents ORFs, shown in different colors indicating their functional categorizes. The GC skew is indicated in the middle circle. The GC content is shown in the innermost circle in black and gray.

Herein, a phage infecting K. pneumoniae 3589, a CRKP strain, was isolated from hospital wastewater collected in Nanjing, China (118.782752 N, 32.078402 W), and designated BUCT-3589. It has an icosahedral head (52.67 ± 1.64 nm) connected to a short tail (14.50 ± 1.48 nm) (Fig. 1A). In a phylogenetic tree constructed using MEGA V7.0 (Fig. 1B), BUCT-3589 shared the same clade as Klebsiella phage Kp Pokalde 002 and *Przondovirus* KP32 (9), which all belong to the genus *Przondovirus* in the family *Autographiviridae*, indicating it as a new member in the same genus. BUCT-3589 possesses a double-stranded DNA genome of 40,757 bp, with a GC content of 53.13%. BLASTn analysis showed that BUCT-3589 shared 91% query coverage and 97% identity with Klebsiella virus KP32 in its genomic sequence. BUCT-3589 encodes 50 ORFs in total, and no toxic, lysogenic, or allergic genes were found in its genome. These ORFs can be categorized into six groups, including lysis, packaging, regulation, replication, structure, and uncharacterized genes (Fig. 1C).

In conclusion, BUCT-3589 has no harmful genes in its genome and can potentially be used as a therapeutic agent to control multidrug-resistant K. pneumoniae strains.

### Data availability.

The complete genome sequence of phage BUCT-3589 was deposited at GenBank under the accession number OP256047. The raw sequence reads of phage BUCT-3589 were deposited in the SRA under the accession number SRR22404838 and the BioProject accession number PRJNA904024.

## References

[1] Han K, Zhu Y, Li F, Li M, An X, Song L, Fan H, Tong Y. 2022. Genomic analysis of bacteriophage BUCT86 infecting Klebsiella pneumoniae. Microbiol Resour Announc 11:e0123821. doi:10.1128/mra.01238-21. 35404092 PMC9119126

[2] Hu Y, Tong S, Li P, An X, Song L, Fan H, Tong Y. 2021. Characterization and genome sequence of the genetically unique Escherichia bacteriophage vB_EcoM_IME392. Arch Virol 166:2505–2520. doi:10.1007/s00705-021-05160-5. 34236511

[3] Li Y, Han P, Pu M, Li F, Li M, An X, Song L, Fan H, Tong Y, Chen Z. 2022. Genomic analysis of the Serratia marcescens bacteriophage BUCT660. Microbiol Resour Announc 11:e0040622. doi:10.1128/mra.00406-22. 35862919 PMC9387219

[4] Han K, He X, Fan H, Song L, An X, Li M, Tong Y. 2022. Characterization and genome analysis of a novel Stenotrophomonas maltophilia bacteriophage BUCT598 with extreme pH resistance. Virus Res 314:198751. doi:10.1016/j.virusres.2022.198751. 35307481

[5] Wei B, Cong C, Zhang L, Zheng L, Chen L, Yu W, Xu Y. 2021. Complete genome analysis of the newly isolated Shigellasonnei phage vB_SsoM_Z31. Arch Virol 166:2597–2602. doi:10.1007/s00705-021-05121-y. 34117533

[6] Liu Y, Mi L, Mi Z, Huang Y, Li P, Zhang X, Tong Y, Bai C. 2016. Complete genome sequence of IME207, a novel bacteriophage which can lyse multidrug-resistant Klebsiella pneumoniae and Salmonella. Genome Announc 4:e01015-16. doi:10.1128/genomeA.01015-16. 27789630 PMC5084854

[7] Bolger AM, Lohse M, Usadel B. 2014. Trimmomatic: a flexible trimmer for Illumina sequence data. Bioinformatics 30:2114–2120. doi:10.1093/bioinformatics/btu170. 24695404 PMC4103590

[8] Bankevich A, Nurk S, Antipov D, Gurevich AA, Dvorkin M, Kulikov AS, Lesin VM, Nikolenko SI, Pham S, Prjibelski AD, Pyshkin AV, Sirotkin AV, Vyahhi N, Tesler G, Alekseyev MA, Pevzner PA. 2012. SPAdes: a new genome assembly algorithm and its applications to single-cell sequencing. J Comput Biol 19:455–477. doi:10.1089/cmb.2012.0021. 22506599 PMC3342519

[9] Tamura K, Stecher G, Peterson D, Filipski A, Kumar S. 2013. MEGA6: molecular evolutionary genetics analysis version 6.0. Mol Biol Evol 30:2725–2729. doi:10.1093/molbev/mst197. 24132122 PMC3840312

